# Heritabilities for the puppy weight at birth in Labrador retrievers

**DOI:** 10.1186/s12917-019-2146-8

**Published:** 2019-11-06

**Authors:** Claude Schelling, Claude Gaillard, Jane Russenberger, Lou Moseley, Gaudenz Dolf

**Affiliations:** 1 Clinic of Reproductive Medicine, Vetsuisse Faculty, University of Zurich, Eschikon 27, 8315 Lindau, Switzerland; 2 Institute of Genetics, Vetsuisse Faculty, University of Bern, Bremgartenstrasse 109a, 3001 Bern, Switzerland; 3 Guiding Eyes for the Blind, 611 Granite Springs Road, Yorktown Heights, NY 10598 USA

**Keywords:** Puppy weight at birth, Predictors, Labrador retriever, Heritabilities, Maternal effects

## Abstract

**Background:**

Weight at birth is an important predictor of neonatal mortality and morbidity in dogs. In addition, the birthweight of the puppies in a litter influences the decision to perform a cesarean section. The goal of the present study was to estimate heritabilities for the puppy birth weight in Labrador retrievers.

**Results:**

Of the 1138 Labrador retriever litters whelped at the Guiding Eye for the Blind between September 2001 and February 2018, 1013 were included in the analyses after data editing. Puppy weight at birth was the target trait, measured on a continuous scale in pounds, and converted to grams. Linear mixed models were used to identify factors influencing puppy weight at birth. The analyses showed that the sex of the puppy, litter size, length of gestation, adult weight of the dam, parity, year of birth and inbreeding coefficient of the puppies and dams contributed to the variance of the puppy birth weight. Dam and litter effects were included as random effects. A multiple trait derivative free restricted maximum likelihood approach was used to estimate variance components and genetic parameters with two animal models, one without covariates (Model 1) and one with covariates (Model 2). Sex of the puppy and litter size had moderate effects, whereas gestation length, adult weight of the dam, parity, year of birth and inbreeding coefficients of the dam and the puppies had minor effects. Estimates for Model 1 and Model 2 were 0.21 and 0.17 for the direct heritabilities, 0.22 and 0.22 for the maternal additive genetic heritabilities, 0.07 and 0.07 for the maternal permanent environmental proportions, and 0.14 and 0.08 for the environmental proportion of the litter.

**Conclusions:**

In order to estimate reliable breeding values for puppy weight at birth, sex of puppy, litter size, length of gestation and the adult weight of the dam should be included. Estimates could benefit from weighing the dams prior to each mating.

## Background

The size or weight of puppies at birth is an important parameter, which influences neonatal mortality and morbidity and is a predictor of puppy survival [1–3]. After a physiological weight loss within the first three days post-partum the growth curve reverses and birth weight is doubled after two weeks [4]. Breeders should weigh the puppies daily for at least the first three weeks [5] and provide each puppy with an individual feeder bowl. In this way, problem puppies are recognized early and the breeder may seek advice from a veterinarian. A recent publication proposes health monitoring for newborn puppies by assessing blood and other parameters to reduce neonatal losses [6].

Factors with an influence on the birthweight have been reviewed for domestic animals [7]. Among them are year and season of birth, sex, age and diet of the dam and fetal environment.

A low birthweight may be caused by a short gestation time and/or intrauterine growth retardation as reported in humans and other mammalian species including the dog [8, 9]. Possible influences on gestation length in dogs including breed, litter size, parental age and size or parity have been investigated in different breeds [2, 10–13], but were not conclusive. This could be due to differences in the assessment and/or the classification of the data. A recent genome-wide association study revealed six canine SNPs associated with gestation length [14], which may lead to the identification of genetic variants influencing this trait.

Birth weight is not only determined by the genetic makeup of the offspring and its environment, but also by the maternal genetic composition and environment provided by the dam [15]. Estimates for genetic parameters for the birth weight in dogs are scarce. Nielen and coworkers [16] estimated the direct heritability for birth weight in Boxers to be 0.62. Helmink and coworkers estimated direct heritabilities for German shepherds (GS) and Labrador retrievers (LR) to be in the range of 0.14 to 0.17 and 0.26 to 0.36, respectively, depending on the model applied [17]. In the same study the maternal additive genetic heritabilities were estimated to be in the range of 0.55 to 0.56 for GS and 0.44 to 0.48 for LR. Estimation of maternal effects may improve breeding value estimation.

Guiding Eyes for the Blind is a non-for profit organization that breeds and trains mostly LR and a few GS to provide guide dogs to people who are blind or have visual impairment. The breeding strategy and detailed procedures of Guiding Eyes for the Blind have been described [18]. The aim of the present study was to identify factors that influence puppy weight at birth which can provide insights for improvement in the Guiding Eyes for the Blind breeding program.

## Results

To reliably estimate parameters levels of covariates with less than 30 litters were not included in the analyses. As a result of this restriction, 91 litters with a litter size smaller than four puppies or larger than eleven puppies, 15 litters with a gestation length shorter than 56 days or longer than 63 days and 19 litters with parities larger than six were dropped, leaving 1013 litters with 7827 puppies in the study. The average puppy weight was 485 g, and males were heavier (497 g) than females (472 g). Adult dam weight, inbreeding coefficient of the dam and puppy, sex, year of birth of the litter, length of gestation, litter size, parity and parity squared were identified to be significant covariates (*p* ≤ 0.05) for PBW and were included in model 2 (Table 1). The fixed effects of season of birth of the litter, inbreeding coefficient of the sire, as well as adult weight of the sire did not have a significant effect on PWB in LR.

**Table 1:** Effects influencing the individual birth weight of puppies. Comparison of the variance components of a model without covariates (Model 1) and a model with covariates (2). The direct-maternal covariance was held at zero

Covariate/Variance	Model 1	Model 2
Adult weight of the dam in kg		5.30
Inbreeding coefficient of the individual		−81.20
Inbreeding coefficient of the dam		101.17
Sex		−24.37
Year of birth		2.16
Length of gestation in days		5.84
Size of the litter		−11.40
Parity		3.95
Parity squared		−4.18
Variance direct genetic	1032.40	657.13
Variance maternal genetic	1088.56	835.16
Variance dam environmental	357.67	273.48
Variance litter environmental	701.47	324.80
Residual	1765.53	1787.46
Total variance	4945.64	3878.04

Sex of the puppy and litter size had moderate effects, whereas gestation length, adult weight of the dam, parity, year of birth and inbreeding coefficients of the dam and the puppies had minor effects (Table 1). Estimates of variance components (Table 1) and genetic parameters (Table 2) for PWB are shown for models 1 and 2. To make the individual effects of the covariates (model 2) more tangible, detailed information about their magnitude are given in Additional file 1.

**Table 2:** Proportions of variance components influencing the individual birth weight of puppies. Comparison of the variance components of a model without covariates (1) and a model with covariates (2). The direct-maternal covariance was held at zero

Covariate/Variance	Model 1	Model 2
Heritability direct	0.21 ± 0.047	0.17 ± 0.041
Heritability maternal	0.22 ± 0.053	0.22 ± 0.049
Dam environmental proportion	0.07 ± 0.034	0.07 ± 0.031
Litter environmental proportion	0.14 ± 0.013	0.08 ± 0.010
Residual proportion	0.36 ± 0.033	0.46 ± 0.032
Total heritability*	0.32	0.28

* $$ {h}_T^2=\left({\sigma}_d^2+0.5\bullet {\sigma}_m^2+1.5\bullet {\sigma}_{dm}\right)/{\sigma}_p^2 $$

The relatively large effect of sex with 24 g in favor of male puppies led to the question: Are we looking here at a case of sexual dimorphism caused by direct and/or maternal additive genetic effects [19]? To clarify this question a bivariate mixed model was applied using WOMBAT [20] with the male PWB as one trait and female PWB as the other one. The aim was to measure the genetic correlation between the two traits (Additional file 2). The genetic correlations of the direct effects as well as the one of the maternal effects reached almost unity. These results indicate that the architecture of the direct as well as the maternal additive genetic effects of PWB should be very similar in both sexes. These findings allowed for jointly analyzing the PWB of both sexes and to run a univariate mixed animal model with sex as covariate.

## Discussion

Birth weight is a complex trait and influenced by many factors. In LR, individual PWB has an influence on the decision if a cesarean section is performed [18]. PWB and post-natal weight gain are important parameters to recognize problem neonates and puppies deviating from normal development, respectively. Variation in size is desirable for Guiding Eyes for the Blind client placements. Dogs provided to people who are blind or visually impaired and also have difficulties with balance require a larger dog to aid in stability. In contrast, many guide dog users prefer a smaller more compact dog to easily fit in smaller spaces available when using commercial means of transportation. However, too heavy or too large guide dogs may be a hazard for the user [17]. Avoiding the birth of puppies with extreme birth weights is desirable in dog breeding and enhances welfare of the animals in general.

In dogs, season of birth was associated with risk for cardiovascular disease risk [21] and fertility in bitches kept in tropic countries [22], however, the authors are not aware of any work reporting a seasonal effect on PBW. In the present study the season in which the LR litters were born did not affect the PBW. This is in contrast to findings in humans where the season of birth was strongly associated with birthweight and adult weight, as well as health outcomes in later life [23]. Seasonal effects on birth weight were found in horse or sheep [24, 25].

Sex of the individual affected birth weight in LR. On average, female puppies were 24 g lighter than male puppies. This effect of the sex of an individual confirms the results of earlier studies in the dog [3, 16, 26] and may reflect physiological differences between the sexes. In our data a genetical sex dimorphism could not be detected. As in full sib families dominance effects could affect the estimation of additive genetic effects [27, 28] we also investigated possible dominance effects in our data using WOMBAT together with the R-package NADIV [27]. With the same approach we also assessed possible sex chromosomal influences usually not considered in variance component analyses. Our data revealed neither substantial dominance effects nor sex-linked effects (Additional file 3).

The antagonistic relation between litter size and PWB is well known for domestic animals [7]. In LR, an increase of the litter size by one puppy resulted in a moderate decrease of PBW of 11 g in average confirming results of earlier studies in the dog [3, 16, 29, 30].

Not surprisingly, longer gestation resulted in an increase of PWB in LR. However, the effect was small with an increase of about 6 g for an additional day in the length of gestation.

Whereas the adult weight of the sire did not influence PWB in LR, offspring of heavier dams showed a slightly higher PWB, on average by 5 g per kg adult weight, which confirms a previous report [3]. In a study of Great Danes with a rather restricted data set, the maternal and paternal adult weight had a positive effect on the PBW. Furthermore, higher adult weight of the sire increased neonatal weight gain in this breed [29].

In LR, up to about parity two to three the PWB in LR increased about 4 g in a linear fashion whereas from about parity two to three to parity six the PWB decreased about 4 g in a non-linear fashion. A similar observation was made in humans [31].

Although the PWB was fluctuating over the years there was a very small increase of 2 g per year from 2001 to 2018. It is well known, that the year of birth may lead to variation of the birth weight by differences in the climate, management and selection of breeding animals in domestic species [7]. The reason for this very small increase of PWB in LR remains unclear but may be related to the selection of breeding animals.

Inbreeding may affect many traits including birth weight [32] and litter size [33] in domestic animal species. In the present study, PBW was only marginally influenced by the inbreeding coefficient. Inbreeding of the puppies and the dam had very small but opposite effects. A 1 % increase in the dam or in the individual resulted in a higher or lower birth weight by 1 g, respectively.

Comparing the total variance of the two models (Table 1) the covariates in model 2 seem to absorb about 20% of the total variance in model 1. The residual variances are not different but the environmental variance of the litter in model 2 is less than half of that in model 1 and the direct genetic variance in model 2 is close to half of that in model 1. The differences of the maternal genetic variance and the environmental variance of the dam are much less pronounced between the two models. These observations are reflected in the estimates of heritabilities and proportions (Table 2). The maternal heritability (0.22 and 0.22) and the environmental proportion of the dam (0.07 and 0.07) are practically identical in both models whereas the direct heritability (0.21 and 0.17) and the environmental proportion of the litter (0.14 and 0.08) are larger in model 1. The residual is larger in model 2 due to the smaller total variance. Total heritability [34] for PWB in LR was 0.32 and 0.28 in model 1 and 2, respectively (Table 2). In Boxers, estimates for heritability of birthweight (corrected for litter effects and sex) were much higher (0.62) [16]. This discrepancy may be explained by the fact that for our study maternal effects were included in the models. Helmink and coworkers estimated heritabilities for birthweight in German shepherd dogs and LR [17] by using the following bivariate models: birth weight – 42 days weight and birth weight - mature weight accounting for the litter. For birthweight in LR they found similar direct genetic heritabilities 0.17 and 0.12, but higher maternal heritabilities 0.35 and 0.37 respectively.

Our results suggest that the inclusion of covariates may lead to better estimates although standard errors of heritabilities and proportions are only marginally smaller in model 2 than in model 1.

## Conclusions

Our findings apply to the colony of LR of the Guiding Eyes for the Blind and are not necessarily meaningful for LR breeding at large. However, the knowledge benefits anyone (especially the working dog community) who wants to investigate birth weight in a specific population, canine or not, or improve the situation with respect to birth weight in specific populations. Results suggest that the inclusion of covariates in the model improves the estimates of variance components. The magnitude of the heritabilities indicates that estimation of breeding values could improve breeding program with respect to PWB. Whether our findings help to improve the situation with respect to the stabilization of PWB in guide dogs depends heavily on how they can be implemented in a breeding strategy that is focused on the guiding abilities of the dogs. To evaluate the impact of our results on the general LR population, reliable data on PWB need to be collected. Most of the covariates included in model 2 could also be recorded in the field.

## Methods

Statistical analyses were carried out using Stata/SE 15.1 (StataCorp, 4905 Lakeway Drive, College Station, Texas 77,845, USA) and MTDFREML [35]. The pedigree provided by Guiding Eyes for the Blind included 10,086 LR. The target trait, individual puppy weight at birth (PWB) was measured in pounds and converted to grams. The final data set comprised 7827 puppies in 1013 litters by 386 dams and 193 sires, born from September 2001 to February 2018 (Additional file 4). Potential predictors were chosen based on literature [4, 11, 26, 29, 36, 37]. Breed, a well-known factor influencing PBW [6] was not relevant for the present study because all animals were LR. The diet of the dam during pregnancy can influence the birth weight of puppies [26], but was not included in the analyses, because keepers of pregnant dams adhere to the feeding regime recommended by the Guiding Eyes for the Blind. Descriptive statistics for PWB and variables in the analyses are given in Additional file 5. Prior to the estimation of variance components, the significance of factors was evaluated and correlations between explanatory variables estimated (Additional file 6). Litter size ranged from 4 to 11. Litters with less than four puppies and litters with twelve or more puppies were excluded from the analyses. Parity (ranging from 1 to 6) and parity squared, as well as length of gestation were included. Length of gestation was calculated between the date of progesterone rise/release of lutenizing hormone plus 5 days and the whelp date measured in days. Litters with gestation lengths shorter than 56 days or longer than 63 days were excluded. Litters after cesarean sections were included in the analyses, because the gestation time was known. Year of birth was a possible predictor and encompassed the years 2001 to 2018. Further possible predictors were the inbreeding coefficients of the puppies and their parents, as well as the sex of the puppies. Finally, adult weight of the dam was measured in pounds then converted to kg.

Using MTDFREML two models were used to estimate variance components of PWB. Model 1 contained no covariates, because covariates are not always easily recorded in the field, and Model 2 included the covariates identified to influence PWB (Additional file 7). The covariance between direct and maternal genetic effects fluctuated around zero and was never different from zero. Therefore, it was fixed at zero for both models. For both models the following variance components were estimated: direct genetic variance, maternal genetic variance, maternal environmental variance, environmental variance by the litter and residual variance.

## Supplementary information

**Additional file 1.** Effects of covariates.

**Additional file 2.** Sex-specific bivariate analyses of male and female PWB.

**Additional file 3.** Estimation of the dominance and sex-linked genetic variance components.

**Additional file 4.** Datafile.

**Additional file 5.** Descriptive statistics of variables.

**Additional file 6.** Statistical Analyses of the birth weight in Labrador Retrievers using Stata.

**Additional file 7.** Animal model of the birth weight in Labrador Retrievers using MTDFREML.

## Data Availability

The datasets used and/or analyzed during the current study are available from the corresponding author on reasonable request.

## Abbreviations

GS: German Shepherd
LR: Labrador retriever
PWB: puppy weight at birth
