# Supplementary material for: Heritabilities for the puppy weight at birth in Labrador retrievers
Source: BMC Vet Res. 2019 Nov 6;15:395. doi: 10.1186/s12917-019-2146-8 (PMC6833269; doi:10.1186/s12917-019-2146-8)
Supplement: Supplementary file 1 — Additional file 1. Effects of covariates. [file 12917_2019_2146_MOESM1_ESM.docx]

**Supplemental file 1 : Individual effects on puppy weight at birth after model 2 (with covariates)**

___________________________________________________________________

**Individual effect** **Increase by** **Effect size (grams)**

___________________________________________________________________

Puppy sex* +24.67

Litter size one puppy -11.40

Length of gestation one day +5.84

Adult weight dam one kilogram +5.30

Parity linear one parity +3.95

Parity quadratic -4.18

Year of birth one year +2.16

Inbreeding coefficient of puppy one percent -0.81

Inbreeding coefficient of dam one percent +1.01

*relative to female puppies
