# Supplementary material for: Heritabilities for the puppy weight at birth in Labrador retrievers
Source: BMC Vet Res. 2019 Nov 6;15:395. doi: 10.1186/s12917-019-2146-8 (PMC6833269; doi:10.1186/s12917-019-2146-8)
Supplement: Supplementary file 2 — Additional file 2. Sex-specific bivariate analyses of male and female PWB. [file 12917_2019_2146_MOESM2_ESM.docx]

# Supplemental file 2: Sex-specific bivariate analyses of male and female PWB

The genetic evaluation revealed a notable difference between male and female puppies in PWB of 24g (see main text). The question arises: Are we looking here at a case of sexual dimorphism? Wolak et al. (2015) demonstrate that if sexual dimorphism is present then a joint evaluation of both sexes would result in a biased estimation of the additive genetic variance components and the heritabilities. To clarify this question a bivariate model was applied (Wolak et al. 2015, van der Heide et al. 2016) where two new phenotypes were created: PWB of male (mPWB) and PWB of female (fPWB). Therefore, male puppies will have missing values in fPWB and female puppies missing values in mPWB. This approach makes it impossible to estimate a residual covariance between the two sexes as no individual can express the trait in both sexes. If the genetic correlation between mPWB and fPWB of direct as well as of maternal effects is unity then both sexes can be modelled jointly to obtain unbiased additive genetic variances. If the genetic correlation is clearly lower than 1, then a univariate model with, “sex” as fixed effect, will produce biased estimate of the additive genetic variances (Wolak et al., 2015).

**Software used for animal models**

Data were analyzed based on linear mixed models using average information REML implemented in the software WOMBAT (Meyer, 2007). Unlike MTDFREML, WOMBAT estimates standard errors of variance components, by Monte Carlo sampling and can easily be combined with other software (see supplemental file 7). In addition, Stata/SE 15.1 was used for graphics and the R package HDInterval for the calculation of the highest density intervals (Kruschke, 2011).

**Models**

**Sex-specific bivariate model**: The dependent variables mPWB and fPWB were analyzed with the same model as Model 2 (see main text) but without the independent variable “Sex”.
By Monte Carlo sampling 10'000 samples of the (co)variance matrix of direct and maternal genetic effects were generated to calculate the 95% highest density intervals (HDI) of the genetic correlation between mPWB and fPWB. The HDI is the interval which contains the required mass such that all points within the interval have a higher probability density than points outside the interval. The advantage of HDI is that it takes into account the fact that the correlation cannot be greater than 1 in our case, whereas the approximate REML SE does not consider any boundary constraints.

**Both sexes jointly modeled in a univariate model**: This model correspond to Model 2 (see main text)

**Results**

**Table 1.** Variances and ratios with the phenotypic variance and their standard errors (SE) respectively as well as covariances and correlations with their SEs for the sex-specific bivariate model of male and female puppy weight at birth.

| **(Co)Variance** | **Variance component** | |  | **Variance ratio** | |
| --- | --- | --- | --- | --- | --- |
|  | **Estimates** | **SE** |  | **Estimates** | **SE** |
| **Direct additive genetic** |  |  |  |  |  |
| mPWB | 723.110 | 189.909 |  | 0.185 | 0.046 |
| mPWB, fPWB | 624.328 | 160.628 |  | 1.000 | 0.031 |
| fPWB | 539.044 | 161.033 |  | 0.141 | 0.040 |
| **Maternal additive genetic** |  |  |  |  |  |
| mPWB | 815.341 | 225.635 |  | 0.208 | 0.052 |
| mPWB, fPWB | 834.012 | 211.225 |  | 1.000 | 0.022 |
| fPWB | 853.115 | 214.445 |  | 0.223 | 0.050 |
| **Permanent maternal environment** |  |  |  |  |  |
| mPWB | 328.899 | 133.280 |  | 0.084 | 0.035 |
| mPWB, fPWB | 274.922 | 115.430 |  | 0.981 | 0.080 |
| fPWB | 238.555 | 117.228 |  | 0.062 | 0.032 |
| **Nonmaternal environmental effects common to littermates** |  |  |  |  |  |
| mPWB | 388.703 | 57.134 |  | 0.099 | 0.015 |
| mPWB, fPWB | 326.432 | 40.546 |  | 1.000 | 0.103 |
| fPWB | 274.140 | 51.348 |  | 0.072 | 0.014 |
| **Residual** |  |  |  |  |  |
| mPWB | 1657.980 | 99.708 |  | 0.424 | 0.035 |
| fPWB | 1918.260 | 91.829 |  | 0.502 | 0.034 |
| **Phenotypic variance** |  |  |  |  |  |
| mPWB | 3914.040 | 175.830 |  | --- | --- |
| mPWB, fPWB | 2059.690 | 194.251 |  | 0.532 | 0.032 |
| fPWB | 3823.110 | 164.517 |  | --- | --- |

mPWB = male puppy weight at birth, fPWB = female puppy weight at birth

mPWB, fPWB = covariance and correlation between male and female puppy weight at birth.

**Table 2.** Variances and ratios with the phenotypic variance and their standard errors (SE) respectively for the univariate model of puppy weight at birth, which correspond to Model 2.

| **(Co)Variance** | **Variance component** | |  | **Variance ratio** | |
| --- | --- | --- | --- | --- | --- |
|  | **Estimates** | **SE** |  | **Estimates** | **SE** |
| **Direct additive genetic** | 658.397 | 165.712 |  | 0.170 | 0.041 |
| **Maternal additive genetic** | 835.919 | 212.441 |  | 0.215 | 0.049 |
| **Permanent maternal environment** | 273.295 | 115.066 |  | 0.070 | 0.031 |
| **Nonmaternal environmental effects common to littermates** | 324.832 | 36.954 |  | 0.084 | 0.010 |
| **Residual** | 1787.060 | 82.752 |  | 0.461 | 0.032 |
| **Phenotypic variance** | 3879.500 | 159.331 |  |  |  |

**Comment:**

- The phenotypic standard deviation (SD) of PWB is same for both sexes in the sex-specific bivariate model (square root of 3914.04 and 3823.11 of Table 1) and also the same as the one obtained with the univariate model (square root of 3879.5 of Table 2), namely 62 g.
- The estimates of covariates of WOMBAT and MTDFREML were very similar (estimates not shown).
- The direct heritability of mPWB (0.18) is 0.04 larger than the one of fPWB (0.14) in the sex-specific bivariate model. The h^2^ of the univariate model with 0.17 is inbetween these two estimates. All SEs lie in between 0.04 and 0.05.
- Heritabilities of maternal genetic effects of the sex-specific bivariate model as well as of the univariate model are almost identical: 0.21 for mPWB, 0.22 for fPWB and 0.22 for PWB, respectively.
- The genetic correlations between mPWB and fPWB were estimated to be 1.0 for both, direct and maternal genetic effects. The 95% HDI of the 10’000 sampled genetic correlations between mPWB and fPWB lie between 0.999994 and 0.999998 for the direct effects and 0.999995 and 0.999999 for the maternal effects, i.e. the credible interval of estimates are very narrow (Figure 1).
- The genetic correlations between mPWB and fPWB of the direct and maternal effects are almost 1 and indicate that the genetic background of PWB may be very similar in males and females. This finding allows analyzing both sexes jointly in a univariate mixed animal model.

**Figure 1**. Distribution of the 10’000 sampled genetic correlations between mPWB and fPWB for direct (a) and maternal (b) additive genetic effects

a b


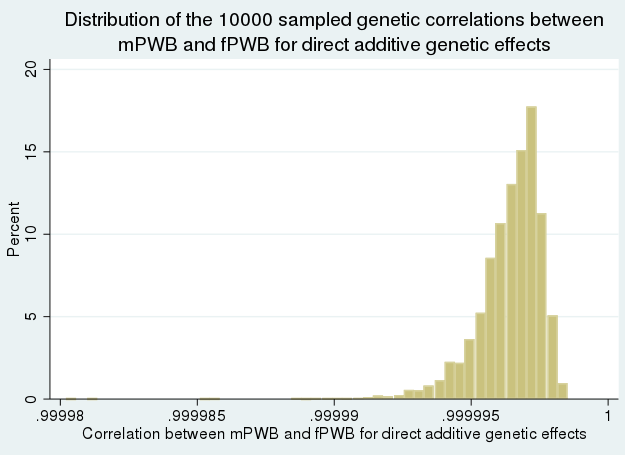

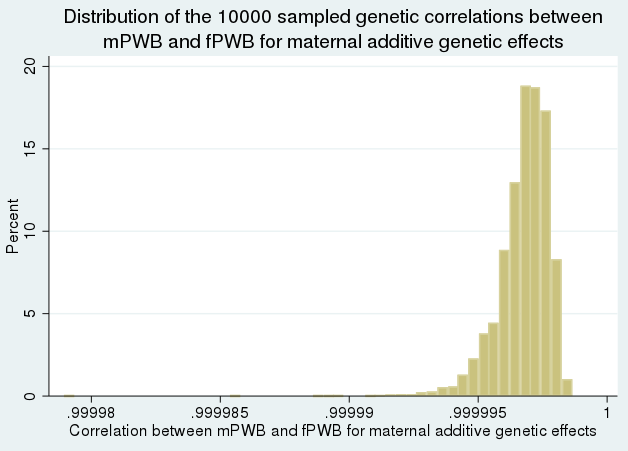


**References**

Kruschke JK. Doing Bayesian data analysis: a tutorial with R and BUGS. Elsevier 2011, Amsterdam, section 3.3.5.

Meyer K, WOMBAT – A tool for mixed model analyses in quantitative genetics by REML. J Zhejiang Uni SCIENCE B 2007; 8: 815–821 doi:10.1631/jzus.2007.B0815.

van der Heide EMM, Lourenco DAL, Chen CY, Herring WO, Sapp RL, Moser DW, Tsuruta S, Masuda Y, Durco BJ, Misztal I. Sexual dimorphism in livestock species selected for economically important traits. J Anim Sci 2016,94: 3684-3692 doi:10.2527/jas2016-0393.

Wolak ME, Roff DA, Fairbairn DJ. Are we underestimating the genetic variances of dimorphic traits? Methods Ecol Evol 2015; 5(3):590-597 doi: 10.1002/ece3.1361.
