# Supplementary material for: Heritabilities for the puppy weight at birth in Labrador retrievers
Source: BMC Vet Res. 2019 Nov 6;15:395. doi: 10.1186/s12917-019-2146-8 (PMC6833269; doi:10.1186/s12917-019-2146-8)
Supplement: Supplementary file 3 — Additional file 3. Estimation of the dominance and sex-linked genetic variance components. [file 12917_2019_2146_MOESM3_ESM.docx]

# Supplemental file 3: Estimation of the dominance and sex-linked genetic variance components

**Introduction**

The phenotypic variance of a quantitative trait can be partitioned into additive genetic, non-additive genetic and environmental sources of variation. The non-additive genetic variance can be further subdivided into dominance and epistatic variances. In most cases dominance and epistasis, are assumed to be of little significance and are included in the residual term of models. If dominance genetic effects are present, but not included in an animal model, they can potentially bias the prediction of the additive genetic effects as well as the estimate of additive genetic variance (Wolak, 2012). Mrode (2005, chapter 9) discusses among other topics the prediction of dominance effects using mixed model methodologies. To our knowledge, to date this approach has not been applied in dogs. However, dogs are well suited for estimating dominance effects, as they are a litter-bearing species that is most often they give birth to full-sibs. In this light, it is reasonable to investigate the possible influence of dominance effects on birth weight in dogs. Wolak (2012) developed an R software package NADIV that contains functions to create and use non-additive genetic relationship matrices in the animal model of quantitative genetics.

Genetic evaluation in livestock usually assumes only autosomal inheritance. However, there is evidence that sex-linked (X-chromosomal) inheritance may contribute to the explanation of the variation of economically important quantitative traits (Fernando and Grossman, 1990). Animal models can be designed to include sex-linked effects in addition to autosomal inheritance e.g. with the software package WOMBAT (Meyer, 2007). The current method to disentangle autosomal and sex-linked sources of additive genetic variance in animal models makes the untenable assumption of no sex chromosomal dosage compensation (Wolak, 2013, chapter 4). In mammals females inactivate large parts of one copy of the X-chromosome, effectively creating a situation similar to the one found in males. This process is called dosage compensation (Straub and Becker, 2007).

**Software used and estimation of variance components**

The R software package PEDANTICS (Morrissey and Wilson, 2009) was used to prepare the pedigree for use with WOMBAT and NADIV.

Data were analyzed based on linear mixed models using average information REML implemented in the software WOMBAT. The R software package NADIV allows constructing the inverses of genetic relationship matrices for dominance (**D^-1^**) and for the sex-linked additive genetic covariance (**S^-1^**) under any model dosage compensation in an animal model. These inverses were then used in WOMBAT, as so called “gin” files, to estimate variance components according to Wolak (2012, supporting information “WOMBAT Tutorial).

**Data**

The inverses **D^-1^** and **S^-1^** of the extended pedigree, which counted 10086 dogs, were successfully generated with NADIV, but they were not accepted by WOMBAT. We reduced the depth of the extended pedigree by taking only dogs of the data file (N=7827, same sample as in the main text). This list of dogs were then edited by the program “fixPedigree” of PEDANTICS, which returns a pedigree in which all individuals that exist as sire and dam are represented by their own record, occurring before the records of their first offspring. The reduced pedigree file counted 7986 dogs. WOMBAT accepted the needed inverses **D^-1^** and **S^-1^**.

The effect of the pedigree reduction was evaluated by using Model 2 (see main text) with two variants, the first with the reduced pedigree (Model CG1) and the second with the extended pedigree (Model S7.1)). Both variants were calculated without dominance and sex-linked random effects ( Table 1).

Two variants of Model 2 with dominance and sex-linked random effects are presented in Table 2. For the first variant (Model S7.3) the inverse of the S-matrix was calculated with no dosage compensation i.e. both chromosomes are active whereas for the second variant (Model S7.4) a random inactivation in females was assumed.

**Results**

**Table 1.** Variance ratios with the phenotypic variance as well as estimates of covariates and their standard errors (SE) respectively of the models with the reduced and with the extended pedigree

|  | **Reduced pedigree** (Model S7.1) | | |  | **Extended pedigree** (Model S7.2) | |
| --- | --- | --- | --- | --- | --- | --- |
| **Maximum log likelihood** | -34563.189 | | |  | -34553.780 | |
| **Variance component** | **Variance ratio ^1^** | | |  | **Variance ratio** | |
|  | **Estimates** | **SE** | |  | **Estimates** | **SE** |
| Direct additive genetic effects of the sire and the dam | 0.142 | 0.036 | |  | 0.170 | 0.041 |
| Maternal additive genetic effects providing suitable environment | 0.193 | 0.047 | |  | 0.215 | 0.049 |
| Permanent maternal environment effects | 0.082 | 0.034 | |  | 0.070 | 0.031 |
| Nonmaternal environmental effects common to littermates | 0.088 | 0.010 | |  | 0.084 | 0.010 |
| Residual effects | 0.494 | 0.029 | |  | 0.461 | 0.032 |
| Phenotypic variance | 3695.990 | 129.331 | |  | 3879.500 | 159.331 |
| **Covariates** (minimum – maximum) | **Puppy weight in g** | |  | | **Puppy weight in g** | |
|  | **Reg coeff ^2^** | **SE** |  | | **Reg coeff** | **SE** |
| Sex (1=male, 2=female) | -24.421 | 1.084 |  | | -24.372 | 1.083 |
| Adult weight of dam (23kg - 41kg) | 5.401 | 0.795 |  | | 5.295 | 0.804 |
| Inbreeding coeff puppy (0% - 18%) | -0.834 | 0.358 |  | | -0.812 | 0.403 |
| Inbreeding coeff dam (0% - 18%) | 1.359 | 0.610 |  | | 1.011 | 0.637 |
| Parity (1 - 6) linear | 4.157 | 0.899 |  | | 3.950 | 0.938 |
| quadratic | -4.097 | 0.428 |  | | -4.179 | 0.434 |
| Year of birth (2001 - 2017) | 1.719 | 0.604 |  | | 2.156 | 0.757 |
| Gestation length (56 days - 63 days) | 5.873 | 1.028 |  | | 5.838 | 1.020 |
| Litter size (4 puppies - 11 puppies) | -11.439 | 0.642 |  | | -11.400 | 0.640 |

**^1^** Variance ratio: Variance component / Phenotypic variance
**^2^** Reg coeff: Regression coefficient

**Comment of Table 1:**

- The phenotypic SD of PWB of the reduced pedigree (61g=square root of 3695.99) is only 1g smaller than the one of the extended pedigree (62g = square root of 3879.5).
- The variance ratios of the random effects of both Model S7.1 and S7.2 are very similar. The absolute differences of variance ratios within source of variation vary between 0.004 and 0.033.
- The regression coefficients and SEs of the covariates of Model S7.1 and S7.2 are very similar, which suggests that there are no significant differences between the reduced and extended pedigrees.
- Based on the above observations, it can be concluded that an evaluation with the reduced pedigree would give very similar results as with the extended pedigree. The reduced pedigree can therefore be used for further analyzes. The number of dogs with the relevant information remained unchanged (N= 7827).

**Table 2.** Variance ratios with the phenotypic variance as well as estimates of covariates and their SEs respectively, in the case of no dosage compensation (Xngdc) and in the case of a random, with respect to parent of origin, inactivation of one sex chromosome in females (Xhori). Both analyses were performed with the reduced pedigree file.

|  | **Dosage compensation** | | | | |
| --- | --- | --- | --- | --- | --- |
|  | **Xngdc** (Model S7.3) | |  | **Xhori** (Model S7.4) | |
| **Maximum log likelihood** | -34562.991 | |  | -34562.992 | |
|  | **Variance ratio ^1^** | |  | **Variance ratio** | |
| **Variance component** | **Estimates** | **SE** |  | **Estimates** | **SE** |
| Direct additive genetic effects of the sire and the dam | 0.142 | 0.036 |  | 0.142 | 0.036 |
| Maternal additive genetic effects providing suitable environment | 0.194 | 0.047 |  | 0.194 | 0.047 |
| Dominance genetic effects | 0.015 | 0.023 |  | 0.015 | 0.023 |
| Sex - linked additive genetic effects | 0.000 | 0.007 |  | 0.000 | 0.005 |
| Permanent maternal environment effects | 0.081 | 0.034 |  | 0.081 | 0.034 |
| Nonmaternal environmental effects common to littermates | 0.086 | 0.011 |  | 0.086 | 0.011 |
| Residual effects | 0.482 | 0.034 |  | 0.482 | 0.034 |
| Phenotypic variance | 3698.430 | 129.981 |  | 3698.380 | 129.795 |

| **Covariates** (minimum – maximum) | **Puppy weight in g** | |  | **Puppy weight in g** | |
| --- | --- | --- | --- | --- | --- |
|  | **Reg coeff ^2^** | **SE** |  | **Reg coeff** | **SE** |
| Sex (1=male, 2=female) | -24.432 | 1.084 |  | -24.432 | 1.084 |
| Adult weight of dam (23kg - 41kg) | 5.398 | 0.796 |  | 5.398 | 0.796 |
| Inbreeding coeff puppy (0% - 18%) | -0.826 | 0.359 |  | -0.826 | 0.359 |
| Inbreeding coeff dam (0% - 18%) | 1.366 | 0.610 |  | 1.366 | 0.610 |
| Parity (1 - 6) linear | 4.159 | 0.900 |  | 4.159 | 0.900 |
| quadratic | -4.100 | 0.428 |  | -4.100 | 0.428 |
| Year of birth (2001 - 2017) | 1.698 | 0.605 |  | 1.698 | 0.605 |
| Gestation length (56 days - 63 days) | 5.873 | 1.028 |  | 5.873 | 1.028 |
| Litter size (4 puppies - 11 puppies) | -11.431 | 0.643 |  | -11.431 | 0.643 |

**^1^** Variance ratio: Variance component / Phenotypic variance
**^2^** Reg coeff: Regression coefficient

**Comment of Table 2:**

- The likelihood ratio tests between Model S7.3 and S7.1 as well as between Model S7.4 and CG1 show no significant differences (P = 0.820 and P = 0.821, respectively.
- The dominance genetic effects contribute with 0.015 very little to the phenotypic variances in Model S7.3 and S7.4, and are not significantly different from zero with an SE of 0.023.
- The sex-linked additive genetic variance is estimated to be zero in both models and does not contribute to the phenotypic variance of PWB.
- Another indication for the lack of influential genes on the X-chromosomes is that the model without sex-linked effects (Model S7.1) has almost the same variance components as the model with sex-linked effects (Model S7.4).
- To sum up, it is justified to remove the variance components “Dominance genetic effects” and “Sex - linked additive genetic effects” from the model.

**References**

Fernando RL, Grossman M. Genetic evaluation with autosomal and X-chromosomal inheritance. Theor Appl Genet 1990;80:75-80; <https://doi.org/10.1007/BF00224018>.

Meyer K. WOMBAT – A tool for mixed model analyses in quantitative genetics by REML. J Zhejiang Uni SCIENCE B 2007; 8: 815–821; <https://doi.org/10.1631/jzus.2007.B0815>.

Morrissey MB, Wilson AJ. PEDANTICS: an R package for pedigree-based genetic simulation and pedigree manipulation, characterization and viewing. Mol Ecol Resour 2010; 10:711-719; <https://doi.org/10.1111/j.1755-0998.2009.02817.x>

Mrode RA. Linear Models for the Prediction of Animal Breeding Values, 2nd ed. Cambridge, MA: CABI Publishing (2005); ISBN 0-85199-000-2.

Straub T, Becker PB. Dosage compensation: the beginning and end of generalization. Nature Rev Genet 2007; 8:47-57; doi:10.1038/nrg201.

Wolak E. nadiv: an R package to create relatedness matrices for estimating non-additive genetic variances in animal models. Methods Ecol Evol 2012; 3:792–796;
<https://doi.org/10.1111/j.2041-210X.2012.00213.x>

Wolak M E. The Quantitative Genetics of Sexual Differences: New Methodologies and an Empiricall Investigation of Sex-Linked, Sex-Specific, Non-Additive, and Epigenetic Effects. UC Riverside, 2013. Retrieved from <https://escholarship.org/uc/item/0sj7571z>
