# Supplementary material for: Heritabilities for the puppy weight at birth in Labrador retrievers
Source: BMC Vet Res. 2019 Nov 6;15:395. doi: 10.1186/s12917-019-2146-8 (PMC6833269; doi:10.1186/s12917-019-2146-8)
Supplement: Supplementary file 5 — Additional file 5. Descriptive statistics of variables. [file 12917_2019_2146_MOESM5_ESM.docx]

**Supplemental file 5: Descriptive statistics of variables**

**1. Target trait**

Birth weight of puppies in Labrador Retrievers. Puppies were weighed in pounds. Their weights were converted to grams prior to the analyses using 1 lb = 453.59237 g.

| Number of observations | 7'827 |
| --- | --- |
| Mean | 484.75 |
| Standard Deviation | 67.27 |
| Minimum | 181.44 |
| Maximum | 754.10 |
| Median | 487.61 |
| Variance | 4525.08 |
| Skewness | -0.27 |
| Kurtosis | 3.73 |

**2. Categorical covariates considered in the analyses**

**2.1 Sex of the puppies.**

Comparison of the mean birth weight of males and females using a two-sample t test with unequal variances:

| Sex | N | Mean | Std Err | Std Dev | 95% Conf Interval | |
| --- | --- | --- | --- | --- | --- | --- |
| male | 3'901 | 497.12 | 1.06 | 66.38 | 495.04 | 499.21 |
| female | 3'926 | 472.45 | 1.05 | 65.89 | 470.39 | 474.51 |
| combined | 7'827 | 484.75 | 0.76 | 67.27 | 483.26 | 486.24 |
| difference |  | 24.67 | 1.50 |  | 21.74 | 27.60 |

t = 16.50, Satterthwaite’s df = 7823.51

| Hypothesis | P-value |
| --- | --- |
| mean(male) – mean(female) = 0 |  |
| mean(male) – mean(female) < 0 | 1.00 |
| mean(male) – mean(female) ≠ 0 | 0.00 |
| mean(male) – mean(female) > 0 | 0.00 |

**2.2 Gestation length in days**

Comparison of the means of the birth weights (g) of the 7'827 puppies.

| Days | N | Mean | Standard Error | 95% Confidence Interval | |
| --- | --- | --- | --- | --- | --- |
| 56 | 76 | 440.53 | 5.76 | 429.24 | 451.84 |
| 57 | 210 | 457.13 | 4.91 | 447.50 | 466.76 |
| 58 | 863 | 473.35 | 2.21 | 469.02 | 477.67 |
| 59 | 2'050 | 482.48 | 1.43 | 479.68 | 485.28 |
| 60 | 3'027 | 485.40 | 1.21 | 483.03 | 487.77 |
| 61 | 1'309 | 495.47 | 1.96 | 491.63 | 499.31 |
| 62 | 256 | 513.41 | 4.23 | 505.12 | 521.71 |
| 63 | 36 | 493.09 | 6.89 | 479.59 | 506.60 |

One-way ANOVA shows that not all of the means are equal (F = 25.96, P > F = 0.00).

**2.3 Parity**

Comparison of the means of the birth weights (g) of the 7'827 puppies.

| Parity | N | Mean | Standard Error | 95% Confidence Interval | |
| --- | --- | --- | --- | --- | --- |
| 1 | 2'490 | 475.06 | 1.28 | 472.56 | 477.56 |
| 2 | 1'984 | 496.55 | 1.45 | 493.71 | 499.40 |
| 3 | 1'493 | 494.35 | 1.81 | 490.80 | 497.89 |
| 4 | 1'099 | 481.92 | 2.13 | 477.75 | 486.10 |
| 5 | 516 | 475.14 | 2.83 | 469.59 | 480.70 |
| 6 | 245 | 462.00 | 4.67 | 452.84 | 471.16 |

One-way ANOVA shows that not all of the means are equal (F = 37.58, P > F = 0.00).

**2.4 Season**

Comparison of the means of the birth weights (g) of the 7'827 puppies.

| Season | N | Mean | Standard Error | 95% Confidence Interval | |
| --- | --- | --- | --- | --- | --- |
| 1 | 1'946 | 487.76 | 1.56 | 484.70 | 490.83 |
| 2 | 1'939 | 486.86 | 1.56 | 483.81 | 489.91 |
| 3 | 1'926 | 480.82 | 1.55 | 477.87 | 483.85 |
| 4 | 2'016 | 483.56 | 1.41 | 480.79 | 486.34 |

One-way ANOVA shows that not all of the means are equal (F = 4.35, P > F = 0.00).

**2.5. Litter size**

Comparison of the means of the birth weights (g) of the 7'827 puppies.

| Size | N | Mean | Standard Error | 95% Confidence Interval | |
| --- | --- | --- | --- | --- | --- |
| 4 | 151 | 539.39 | 5.67 | 528.28 | 550.50 |
| 5 | 445 | 523.14 | 3.48 | 516.33 | 529.95 |
| 6 | 741 | 508.49 | 2.62 | 503.36 | 513.62 |
| 7 | 1'175 | 501.86 | 1.88 | 498.16 | 505.55 |
| 8 | 1'714 | 483.85 | 1.52 | 480.86 | 486.83 |
| 9 | 1'653 | 476.58 | 1.57 | 473.50 | 479.66 |
| 10 | 1'359 | 459.23 | 1.67 | 455.95 | 462.50 |
| 11 | 589 | 462.16 | 2.27 | 457.71 | 466.62 |

One-way ANOVA shows that not all of the means are equal (F = 109.66, P > F = 0.00).

**2.6. Year of birth**

Comparison of the means of the birth weights (g) of the 7'827 puppies.

| Year | N | Mean | Standard Error | 95% Confidence Interval | |
| --- | --- | --- | --- | --- | --- |
| 2001 | 93 | 483.10 | 7.48 | 468.43 | 497.77 |
| 2002 | 424 | 471.80 | 3.57 | 464.81 | 478.80 |
| 2003 | 542 | 474.52 | 2.95 | 468.75 | 480.30 |
| 2004 | 554 | 474.06 | 2.98 | 468.22 | 479.90 |
| 2005 | 512 | 467.17 | 2.74 | 461.81 | 472.54 |
| 2006 | 577 | 475.74 | 2.73 | 470.40 | 481.08 |
| 2007 | 425 | 465.15 | 3.17 | 458.94 | 471.35 |
| 2008 | 425 | 462.31 | 3.19 | 456.07 | 468.56 |
| 2009 | 398 | 487.58 | 3.55 | 480.61 | 494.54 |
| 2010 | 392 | 484.95 | 2.95 | 479.16 | 490.74 |
| 2011 | 439 | 498.42 | 3.05 | 492.45 | 504.39 |
| 2012 | 448 | 492.18 | 2.92 | 486.45 | 497.90 |
| 2013 | 500 | 489.98 | 2.70 | 484.70 | 495.27 |
| 2014 | 483 | 496.11 | 2.77 | 490.68 | 501.55 |
| 2015 | 464 | 493.33 | 3.33 | 486.81 | 499.86 |
| 2016 | 520 | 501.53 | 2.93 | 495.79 | 507.27 |
| 2017 | 561 | 512.12 | 2.68 | 506.86 | 517.37 |
| 2018 | 70 | 519.93 | 7.80 | 504.65 | 535.21 |

One-way ANOVA shows that not all of the means are equal (F = 22.41, P > F = 0.00).

**3. Continuous covariates considered in the analyses**

**3.1 Adult weight of the sires**

Adult sires were weighed in pounds. Their weights were converted to kilograms prior to the analyses using 1 lb = 0.45359237 kg.

| Number of observations | 7'586 |
| --- | --- |
| Mean | 33.65 |
| Standard Deviation | 2.83 |
| Minimum | 25.85 |
| Maximum | 40.82 |
| Median | 33.11 |
| Variance | 7.99 |
| Skewness | 0.57 |
| Kurtosis | 2.88 |

**3.2 Adult weight of the dams**

Adult dams were weighed in pounds. Their weights were converted to kilograms prior to the analyses using 1 lb = 0.45359237 kg.

| Number of observations | 7827 |
| --- | --- |
| Mean | 28.61 |
| Standard Deviation | 2.49 |
| Minimum | 23.13 |
| Maximum | 40.82 |
| Median | 28.58 |
| Variance | 6.18 |
| Skewness | 0.86 |
| Kurtosis | 4.78 |

**3.3 Inbreeding coefficient of the individuals**

| Number of observations | 7'827 |
| --- | --- |
| Mean | 0.1072 |
| Standard Deviation | 0.0409 |
| Minimum | 0.0000 |
| Maximum | 0.1830 |
| Median | 0.1130 |
| Variance | 0.0017 |
| Skewness | -0.6288 |
| Kurtosis | 3.0285 |

**3.4 Inbreeding coefficient of the sires**

| Number of observations | 7827 |
| --- | --- |
| Mean | 0.0913 |
| Standard Deviation | 0.0425 |
| Minimum | 0.0000 |
| Maximum | 0.1810 |
| Median | 0.0940 |
| Variance | 0.0018 |
| Skewness | -0.2554 |
| Kurtosis | 2.6150 |

**3.5 Inbreeding coefficient of the dams**

| Number of observations | 7827 |
| --- | --- |
| Mean | 0.0942 |
| Standard Deviation | 0.0408 |
| Minimum | 0.0000 |
| Maximum | 0.1820 |
| Median | 0.0940 |
| Variance | 0.0017 |
| Skewness | -0.0832 |
| Kurtosis | 2.5644 |
