# Supplementary material for: Heritabilities for the puppy weight at birth in Labrador retrievers
Source: BMC Vet Res. 2019 Nov 6;15:395. doi: 10.1186/s12917-019-2146-8 (PMC6833269; doi:10.1186/s12917-019-2146-8)
Supplement: Supplementary file 6 — Additional file 6. Statistical Analyses of the birth weight in Labrador Retrievers using Stata. [file 12917_2019_2146_MOESM6_ESM.docx]

**Supplemental file 6: Statistical Analyses of the birth weight in Labrador Retrievers using Stata**

Statistical analyses were carried out using Stata/SE 15.1 (StataCorp, 4905 Lakeway Drive, College Station, Texas 77845, USA). The data set comprised observations of 7'827 individuals of LR from September 2001 to February 2018 by 386 different dams and 193 sires. If not stated differently the significance level for statistical tests is 0.05.

The target trait (**PUPPYWg**), the birth weight of the puppies, was continuous, measured in pounds, and converted to grams prior to the analyses using 1 lb = 453.59237 g.

The following variables were evaluated as covariates and possible predictors for PUPPYWg: gender (**SEX**), year of birth (**BYEAR**), whelping season (**SEASON**), parity (**PARITY**), gestation length (**GESTATION**), litter size (**LSIZE**), weight of the sire (**SIREW**), weight of the dam (**DAMW**), inbreeding coefficient of the individual (**FIID**), inbreeding coefficient of the sire (**FSID**) and inbreeding coefficient of the dam (**FDID**). All variables are described in Supplement 1.

In addition to the variables mentioned above the identities of the dams (**DID**) and the litters (**LID**) were analyzed as random effects.

**1. Correlations**

Correlation coefficients between possible predictors were calculated to avoid the inclusion of highly correlated predictors.

Continuos variables (Stata command: **correlate**):

|  | SIREW | DAMW | FIID | FSID | FDID |
| --- | --- | --- | --- | --- | --- |
| SIREW | 1.00 |  |  |  |  |
| DAMW | 0.07 | 1.00 |  |  |  |
| FIID | - 0.23 | - 0.17 | 1.00 |  |  |
| FSID | - 0.30 | - 0.16 | 0.67 | 1.00 |  |
| FDID | - 0.12 | - 0.19 | 0.55 | 0.29 | 1.00 |

Correlations are moderate with the exception of the elevated correlations between FIID and FSID or FDID. This is not so much surprising as we have a rather closed colony.

Categorical variables with more than two levels (Stata command: **ktau**):

|  | BYEAR | SEASON | PARITY | GESTATION | LSIZE |
| --- | --- | --- | --- | --- | --- |
| BYEAR | 0.94 |  |  |  |  |
| SEASON | - 0.05 | 0.75 |  |  |  |
| PARITY | - 0.07 | 0.00 | 0.77 |  |  |
| GESTATION | 0.15 | - 0.05 | - 0.01 | 0.74 |  |
| LSIZE | - 0.00 | - 0.01 | - 0.05 | - 0.07 | 0.84 |

All correlations are small to very small.

Continuous variables versus categorical variables (Stata command: **ktau**):

|  | BYEAR | SEASON | PARITY | GESTATION | LSIZE |
| --- | --- | --- | --- | --- | --- |
| SIREW | - 0.18 | 0.03 | - 0.00 | - 0.03 | 0.01 |
| DAMW | - 0.19 | - 0.01 | 0.10 | - 0.03 | 0.12 |
| FIID | 0.45 | - 0.01 | - 0.07 | 0.07 | - 0.03 |
| FSID | 0.46 | - 0.02 | - 0.05 | 0.09 | - 0.01 |
| FDID | 0.46 | 0.01 | - 0.16 | 0.08 | - 0.02 |

All correlations are small to very small with the exception of those between BYEAR and the inbreeding coefficients. This is not so much surprising as we have a rather closed colony.

**2. Evaluation of random effects**

To evaluate whether the litter, the dam or the sire, or any combination of them, influence PUPPYWg, corresponding models were analyzed using mixed effects linear regression (Stata command: **mixed**) and compared with the empty model (Stata commands: **estimates store** and **esttab**). This approach was chosen because common information criterions are not permissible in mixed models. Therefore, we wanted to identify a model or models that clearly are better than other models prior to including covariates.

| Model | Structure | n | df | AIC | BIC |
| --- | --- | --- | --- | --- | --- |
| Intonly | Intercept only | 7827 | 2 | 88098.0 | 88111.9 |
| Lit | LID | 7827 | 3 | 84814.9 | 84835.8 |
| Sir | SID | 7827 | 3 | 87405.5 | 87426.4 |
| Dam | DID | 7827 | 3 | 85225.7 | 85246.6 |
| DamLit | DID and LID | 7827 | 4 | 84503.3 | 84531.2 |
| SirLit | SID and LID | 7827 | 4 | 84785.9 | 84813.7 |
| SirDam | SID and DID | 7827 | 4 | 84778.4 | 84806.3 |
| SirDamLit | SID and DID and LID | 7827 | 5 | 84780.0 | 84814.8 |

The model with DID and LID as random effects by far fits the data best. Therefore, covariates were only evaluated in the model with DID and LID a random effects.

**3 Model evaluation for PUPPYWg**

Models were calculated with the restriction **if SIREW != .** as there were missing values (Stata command: **mixed**). In addition to the covariates already mentioned PARITYsq, the parity squared, was added to the full model because the average birth weight first increases and then decreases with increasing parity (see Supplement 1).

**Model m1** (full model)

Number of observations: 7'586

| Covariate | Coefficient | Standard error | z | P > \|z\| | 95% Confidence interval | |
| --- | --- | --- | --- | --- | --- | --- |
| SEX | -24.24 | 1.11 | - 21.86 | 0.00 | - 26.41 | - 22.07 |
| BYEAR | 2.47 | 0.51 | 4.98 | 0.00 | 1.48 | 3.47 |
| SEASON | 0.10 | 0.89 | 0.11 | 0.92 | - 1.65 | 1.84 |
| PARITY | 25.03 | 2.81 | 8.91 | 0.00 | 19.53 | 30.54 |
| PARITYsq | - 4.24 | 0.46 | - 9.24 | 0.00 | - 5.14 | - 3.34 |
| GESTATION | 5.64 | 1.05 | 5.35 | 0.00 | 3.58 | 7.71 |
| LSIZE | - 11.14 | 0.66 | - 16.81 | 0.00 | - 12.44 | - 9.84 |
| SIREW | 0.46 | 0.38 | 1.22 | 0.22 | - 0.28 | 1.20 |
| DAMW | 5.50 | 0.78 | 7.07 | 0.00 | 3.98 | 7.03 |
| FIID | - 119.12 | 45.24 | - 2.63 | 0.01 | - 207.79 | -30.46 |
| FSID | 12.13 | 39.50 | 0.31 | 0.76 | - 65.30 | 89.56 |
| FDID | 212.75 | 60.54 | 3.51 | 0.00 | 94.08 | 331.41 |

| Random effect | Estimate | Standard error | 95% Confidence interval | |
| --- | --- | --- | --- | --- |
| DID | 1007.39 | 99.11 | 830.71 | 1221.64 |
| LID | 419.68 | 41.24 | 346.15 | 508.82 |
| Residual | 2109.15 | 36.74 | 2038.36 | 2182.40 |

For the next model SEASON was dropped.

**Model m2**

Number of observations: 7'586

| Covariate | Coefficient | Standard error | z | P > \|z\| | 95% Confidence interval | |
| --- | --- | --- | --- | --- | --- | --- |
| SEX | -24.24 | 1.11 | - 21.86 | 0.00 | - 26.41 | - 22.07 |
| BYEAR | 2.47 | 0.50 | 4.92 | 0.00 | 1.49 | 3.45 |
| PARITY | 25.04 | 2.81 | 8.92 | 0.00 | 19.54 | 30.54 |
| PARITYsq | - 4.24 | 0.46 | - 9.24 | 0.00 | - 5.14 | - 3.34 |
| GESTATION | 5.64 | 1.05 | 5.35 | 0.00 | 3.57 | 7.70 |
| LSIZE | - 11.14 | 0.66 | - 16.81 | 0.00 | - 12.44 | - 9.84 |
| SIREW | 0.46 | 0.38 | 1.23 | 0.22 | - 0.28 | 1.20 |
| DAMW | 5.50 | 0.78 | 7.07 | 0.00 | 3.97 | 7.02 |
| FIID | - 119.20 | 45.23 | - 2.64 | 0.01 | - 207.86 | -30.54 |
| FSID | 12.20 | 39.50 | 0.31 | 0.76 | - 65.22 | 89.62 |
| FDID | 213.41 | 60.22 | 3.54 | 0.00 | 95.37 | 331.44 |

| Random effect | Estimate | Standard error | 95% Confidence interval | |
| --- | --- | --- | --- | --- |
| DID | 1007.18 | 99.08 | 830.57 | 1221.35 |
| LID | 419.76 | 41.24 | 346.23 | 508.89 |
| Residual | 2109.15 | 36.74 | 2038.36 | 2182.40 |

For the next model FSID was dropped.

**Model m3**

Number of observations: 7'586

| Covariate | Coefficient | Standard error | z | P > \|z\| | 95% Confidence interval | |
| --- | --- | --- | --- | --- | --- | --- |
| SEX | -24.24 | 1.11 | - 21.86 | 0.00 | - 26.41 | - 22.06 |
| BYEAR | 2.50 | 0.49 | 5.12 | 0.00 | 1.54 | 3.46 |
| PARITY | 25.07 | 2.81 | 8.93 | 0.00 | 19.57 | 30.57 |
| PARITYsq | - 4.25 | 0.46 | - 9.27 | 0.00 | - 5.15 | - 3.35 |
| GESTATION | 5.64 | 1.05 | 5.35 | 0.00 | 3.57 | 7.70 |
| LSIZE | - 11.15 | 0.66 | - 16.84 | 0.00 | - 12.45 | - 9.85 |
| SIREW | 0.45 | 0.37 | 1.19 | 0.23 | - 0.29 | 1.18 |
| DAMW | 5.50 | 0.78 | 7.08 | 0.00 | 3.98 | 7.03 |
| FIID | - 110.82 | 36.20 | - 3.06 | 0.00 | - 181.78 | -39.87 |
| FDID | 209.86 | 59.12 | 3.55 | 0.00 | 93.97 | 325.74 |

| Random effect | Estimate | Standard error | 95% Confidence interval | |
| --- | --- | --- | --- | --- |
| DID | 1007.52 | 99.10 | 830.86 | 1221.74 |
| LID | 419.74 | 41.24 | 346.22 | 508.88 |
| Residual | 2109.15 | 36.74 | 2038.36 | 2182.40 |

For the next model SIREW was dropped.

**Model m4**

Number of observations: 7'586

| Covariate | Coefficient | Standard error | z | P > \|z\| | 95% Confidence interval | |
| --- | --- | --- | --- | --- | --- | --- |
| SEX | -24.24 | 1.11 | - 21.86 | 0.00 | - 26.41 | - 22.06 |
| BYEAR | 2.45 | 0.49 | 5.03 | 0.00 | 1.49 | 3.40 |
| PARITY | 25.19 | 2.81 | 8.97 | 0.00 | 19.69 | 30.69 |
| PARITYsq | - 4.27 | 0.46 | - 9.32 | 0.00 | - 5.17 | - 3.37 |
| GESTATION | 5.64 | 1.05 | 5.35 | 0.00 | 3.58 | 7.71 |
| LSIZE | - 11.16 | 0.66 | - 16.85 | 0.00 | - 12.46 | - 9.87 |
| DAMW | 5.51 | 0.78 | 7.08 | 0.00 | 3.98 | 7.03 |
| FIID | - 117.18 | 35.84 | - 3.27 | 0.00 | - 187.42 | -46.94 |
| FDID | 212.97 | 59.11 | 3.60 | 0.00 | 97.12 | 328.82 |

| Random effect | Estimate | Standard error | 95% Confidence interval | |
| --- | --- | --- | --- | --- |
| DID | 1008.89 | 99.25 | 831.97 | 1223.44 |
| LID | 420.92 | 41.31 | 347.27 | 510.19 |
| Residual | 2109.11 | 36.74 | 2038.32 | 2182.36 |

| Model | Structure | n | df | AIC | BIC |
| --- | --- | --- | --- | --- | --- |
| m1 | full | 7586 | 16 | 81087.18 | 81198.12 |
| m2 | SEASON dropped | 7586 | 15 | 81085.19 | 81189.20 |
| m3 | FSID dropped | 7586 | 14 | 81083.28 | 81180.36 |
| m4 | SIREW dropped | 7586 | 13 | 81082.71 | 81172.85 |

Both, AIC and BIC favor Model m4 although the AIC distinctions are marginal.

Since SIRW was dropped we now can use the full 7'827 observations.

**Final model**

Number of observations: **7'827**

| Covariate | Coefficient | Standard error | z | P > \|z\| | 95% Confidence interval | |
| --- | --- | --- | --- | --- | --- | --- |
| SEX | -24.28 | 1.09 | - 22.35 | 0.00 | - 26.41 | - 22.15 |
| BYEAR | 2.37 | 0.48 | 4.96 | 0.00 | 1.44 | 3.31 |
| PARITY | 25.31 | 2.76 | 9.18 | 0.00 | 19.91 | 30.71 |
| PARITYsq | - 4.29 | 0.45 | - 9.47 | 0.00 | - 5.18 | - 3.40 |
| GESTATION | 5.43 | 1.04 | 5.23 | 0.00 | 3.39 | 7.46 |
| LSIZE | - 11.11 | 0.65 | - 16.98 | 0.00 | - 12.39 | - 9.82 |
| DAMW | 5.58 | 0.78 | 7.16 | 0.00 | 4.05 | 7.10 |
| FIID | - 84.18 | 28.94 | - 2.91 | 0.00 | - 140.90 | -27.46 |
| FDID | 203.70 | 58.17 | 3.50 | 0.00 | 89.69 | 317.70 |

| Random effect | Estimate | Standard error | 95% Confidence interval | |
| --- | --- | --- | --- | --- |
| DID | 1017.26 | 98.92 | 840.74 | 1230.86 |
| LID | 425.73 | 40.39 | 353.50 | 512.72 |
| Residual | 2089.44 | 35.83 | 2020.39 | 2160.85 |

**Note**: LID is nested within DID.

Get the standardized residuals of the model (Stata command: **predict**)

| Variable | Observations | Mean | Standard deviation | Minimum | Maximum |
| --- | --- | --- | --- | --- | --- |
| rs | 7827 | - 3.13e-10 | 0.95 | - 6.49 | 5.33 |

The goodness of fit of the model can be assessed by checking whether the standardized residuals are normally distributed. A normal quantile plot (Stata command: **qnorm**) is more sensitive to deviances in the tails.

Check for deviances near the mean of the distribution of the standardized residuals (Stata command: **pnorm**):

Show the fit of the standardized residuals to the standard normal in a density probability plot (Stata command: **dpplot**). The deviances in both, the normal quantile plot and the standardized normal probability plot, are reflected in the density probability plot.

The red curve shows the distribution of the standardized residuals and the blue curve the corresponding normal.
