# Supplementary material for: Heritabilities for the puppy weight at birth in Labrador retrievers
Source: BMC Vet Res. 2019 Nov 6;15:395. doi: 10.1186/s12917-019-2146-8 (PMC6833269; doi:10.1186/s12917-019-2146-8)
Supplement: Supplementary file 7 — Additional file 7. Animal model of the birth weight in Labrador Retrievers using MTDFREML. [file 12917_2019_2146_MOESM7_ESM.docx]

**Supplemental file 7: Animal model of the birth weight in Labrador Retrievers using MTDFREML**

Variance components for the target trait (PUPPYWg), the birth weight of the puppies, were estimated using the program package MTDFREML (Boldman KG, Kriese LA, Van Vleck LD, Van Tassel CP, Kachman SD. A manual for use of MTDFREML. A set of programs to obtain estimates of variances and covariances [DRAFT]. 1995; USDA, Agricultural Research Service).

Model 1 (no covariates)

MTDF66Model01

MTDF76Model01

Model 2 (all coavariates)

MTDF66Model03

MTDF76Model03

MTDF77Model03

-----------------------------------------------------------------------------------------------------------------

Two models were used, Model 1 without any covariates and Model 2 with all covariates identified with Stata (see Supplement 2). In both models, the following variance components were estimated:

• Direct additive genetic variance: Correspond to the predictor “animal” of the model.

• Maternal additive genetic variance: This component captures maternal genetic effects such as the nutrition of the fetuses, the available space in the uterus and the intra- uterine environment.

• Maternal environmental variance: This component captures maternal environmental effects such as the keeping or the health of the dam.

• Environmental variance of the litter: This component captures litter effects caused by the full sibs such as number, sex or health status.

• Residual variance: Anything that has not been accounted for in the model.

The correlation between direct and maternal genetic effects could not reliably be estimated in our data, they reached 0.077 with a SE of 0.188 for Model 1 and -0.052 with a SE of 0.184 for Model 2. Therefore, in both models, the covariance was fixed at zero. The maternal environmental variance and the environmental variance of the litter were assumed to be uncorrelated.

MTDFREML produces several output files. Estimates for the variance components are written to MTDF76. Regression coefficients for the covariates are written to MTDF77. Heritabilities and proportions of variance components are written to MTDF76.
